# A chain mediating model of the impact of physical exercise on sleep quality

**DOI:** 10.1186/s12889-025-22728-x

**Published:** 2025-04-22

**Authors:** Zhilei Zhang, Xiujuan Liu

**Affiliations:** 1 School of Physical Education and Health, Heze University, Heze, China; 2 School of Political Science and Law, Heze University, Heze, China

**Keywords:** College students, Sleep, Physical exercise, Mobile phone dependence, Relationship

## Abstract

**Background:**

In the fast-paced modern life, sleep quality has become a key indicator for measuring individual health and quality of life. The mechanisms by which physical exercise influences sleep quality through psychological and behavioral pathways, particularly via smartphone dependence, remain underexplored.

**Objective:**

To investigate the mediating roles of mobile phone dependence and sleep duration in the relationship between physical exercise and sleep quality, using a chain mediation model.

**Methods:**

A questionnaire survey was conducted on 1905 college students, and data were processed using Pearson correlation analysis and structural equation modeling.

**Results:**

Mobile phone dependence significantly mediated the relationship between physical exercise and sleep quality, with an effect size of 0.036 (*p* < 0.05), accounting for 27% of the total effect. Additionally, mobile phone dependence and sleep duration jointly mediated the relationship, with an effect size of 0.013 (*p* < 0.05), accounting for 10% of the total effect.

**Conclusion:**

Physical exercise not only directly improves sleep quality but also indirectly promotes the improvement of sleep quality by reducing mobile phone dependence and increasing sleep duration. This study innovatively identifies a chain mediation mechanism, offering empirical support for designing integrated interventions targeting both behavioral addiction and sleep physiology in college populations.

## Background

In the fast-paced modern society, sleep quality has become a critical indicator of individual health and quality of life. Among various populations, college students represent a particularly vulnerable group, as their sleep quality directly impacts academic performance, mental health, and long-term career development. Alarmingly, over 40% of college students exhibit clinically significant sleep disturbances-a prevalence substantially higher than that observed in the general population [1]. While insufficient physical activity among adolescents remains a global public health concern [2]; physical activity has been proven to help improve sleep [3], and participation in exercise becomes one of the important factors affecting sleep quality [4]. Physical exercise, a subset of physical activity, is structured, planned, and repetitive, with the objective of improving or maintaining physical fitness (e.g., running, strength training, aerobic exercises). Therefore, the relationship between college students’ physical exercise and sleep quality is worth studying.

According to the 53rd Statistical Report on the Development of the Internet in China released by the China Internet Network Information Center (CNNIC) in Beijing, by December 2023, the scale of netizens in China reached 1.092 billion, with an Internet penetration rate of 77.5%, and the average daily Internet usage time per person was 3.7 h. Some scholars define prolonged use of mobile phones as using mobile phones for more than four hours per day, which can lead to mobile phone dependence, also known as mobile phone addiction or irrational mobile phone use [5], is described as a state of obsession caused by specific motives, long-term use of mobile phones, and damage to individual psychological and social functions. College students, as the main users of smartphones, exhibit particularly noticeable dependence. Previous studies have shown that negative behaviors (such as mobile phone dependence) are closely related to sleep quality [6]. College students’ mobile phone usage behavior has a significant impact on sleep quality; the higher the frequency and longer the duration of mobile phone use before sleep, the worse the sleep quality [7]. In addition to mobile phone dependence, a significant factor affecting college students’ sleep quality is the widespread problem of insufficient sleep [8], which may be related to factors such as mobile phone dependence. A systematic review of 23 randomized controlled trials indicates that exercise intervention may help reduce smartphone addiction [9]. These findings collectively position mobile phone dependence and sleep duration as critical mediators in the relationship between physical exercise and sleep quality.

### The impact of physical exercise on sleep quality

The biopsychosocial model provides a comprehensive framework for understanding sleep health through the dynamic interaction of physiological, psychological, and social determinants [10]. Specifically, physical exercise improves sleep quality through the synergistic effects of different pathways: physiological adaptations; psychological mechanisms such as strengthened self-regulation capacities that redirect attention from maladaptive behaviors (e.g., smartphone overuse) to health-promoting activities; and social reinforcement through group-based exercise environments that foster behavioral consistency [11]. This tripartite mechanism challenges reductionist biomedical paradigms by demonstrating that sleep outcomes fundamentally depend on integrated biological-behavioral-social processes.

A substantial body of research indicates that exercise can improve sleep quality across various populations. Exercise primarily modulates negative emotions such as tension and anxiety, inhibits excessive arousal states, and both aerobic training and combined aerobic and resistance training can enhance sleep quality in patients with primary insomnia [12]. Aerobic combined with resistance exercise intervention can improve sleep quality in gastric cancer chemotherapy patients [13]. Aerobic exercise training significantly improves lung function and sleep quality in patients with stable chronic obstructive pulmonary disease [14]. Home-based aerobic exercise can improve sleep disturbances and quality of life in liver cancer patients [15]. Sedentary behavior is a risk factor for sleep disorders; an investigation and analysis of sedentary and exercise behaviors in 23,000 subjects show that exercise is effective in improving sleep disorder symptoms in sedentary groups [16]. Studies have shown that chronic fatigue syndrome patients significantly improved sleep disturbances after three months of fitness Qigong practice [17]. Comprehensive non-pharmacological interventions, including physical exercise and dietary management, for sleep disorder patients have a positive impact on sleep quality, general physical symptoms, and some physical examination indicators of sleep disorders [18]. Exercise intervention in cancer patients after treatment for three months resulted in longer sleep duration, which has significant clinical implications [19]. Exercise intervention in urban elderly insomnia patients led to the subjects sleeping “better” with higher sleep quality [20]. Multiple meta-analyses have shown the effectiveness of exercise intervention on sleep quality. Twenty-two randomized controlled trials included in the analysis showed that self-reported sleep quality, insomnia severity, and daytime sleepiness can be improved or improved through exercise treatment [21]. In 23 high-quality trials, including data from 1,269 patients who received exercise intervention and 1,203 patients who received medication treatment or no intervention (control), meta-analysis showed that exercise intervention has a significant effect on treating primary insomnia, and exercise intervention has a significant positive impact on primary insomnia, especially for elderly patients [22]. A total of 12 randomized controlled trials involving 1,493 subjects were included; exercise intervention programs included yoga, walking, fitness Qigong, and aerobic exercise, and meta-analysis showed that exercise effectively improves sleep in peri-menopausal women, having a significant impact on sleep quality and insomnia symptoms in peri-menopausal women [23]. Based on these extensive studies on the impact of physical exercise on sleep quality, Hypothesis H1 is proposed: Physical exercise has a positive impact on enhancing sleep quality.

### The impact of physical exercise on mobile phone dependence

Self-regulation theory provides a robust theoretical foundation for understanding how physical exercise mitigates mobile phone dependence. According to Zimmerman’s cyclical model of self-regulated learning (SRL), structured exercise regimens—characterized by goal-setting, self-monitoring, and reflective adaptation—strengthen executive functioning and impulse control [24]. The enhancement of self-regulation capacity triggered by exercise effectively reduces the impulse associated with the reward mechanism of smartphones; each increment in physical exercise directly displaces screen time, thereby weakening the triggers of dependence. This process not only regulates individual behavioral motivations from a psychological perspective but also reduces contact with mobile phones at the behavioral level, thus exerting a dual inhibitory effect on mobile phone dependence.

Engaging in physical exercise provides an opportunity to reduce exposure to smartphones, potentially decreasing mobile phone dependence. The issue of mobile phone dependence due to prolonged use should not be overlooked, as it can severely impact one’s physical and mental health [25]. Studies have found that the rate of mobile phone addiction (MPAI) among college students in China is 23% [26]. Prolonged and frequent use of the internet and smartphones often results in a sedentary lifestyle, and research indicates a significant negative correlation between physical exercise and mobile phone dependence [27, 28]. Increasing the frequency of college students’ participation in physical activities each week is equivalent to reducing their habitual long periods of sedentary behavior in daily life, replacing it with a healthier lifestyle that less frequently involves the use of mobile phones [29]. A survey of 650 Chinese college students showed that higher levels of physical activity in terms of aerobic endurance are associated with lower degrees of mobile phone dependence [30]. A 12-week experiment demonstrated that group basketball and Baduanjin exercises reduced smartphone dependence, and this effect continued to be valid for two months [31]. Open and group-based sports had similar effects on improving college students’ mobile phone dependence, while closed sports showed a more sustained effect [32]. Physical exercise can serve as an intervention for mobile phone dependence among college students [33]. Neurobiological evidence suggests that exercise can reduce internet addiction by improving the morphology of specific parts of the central nervous system; protecting the autonomic nervous system; and controlling reward impulses, meaning that exercise seems to alleviate internet addiction by regulating the central and autonomic nervous systems [34]. Based on this, Hypothesis H2 is proposed: Physical exercise has a positive impact on reducing mobile phone dependence.

### The impact of mobile phone dependence on sleep quality

Self-regulation theory posits that an individual’s behavior is influenced by internal motivations and external environmental factors [35]. Prolonged smartphone use before bedtime induces cognitive overload through information saturation and emotional arousal, delaying sleep onset and thereby disrupting normal sleep patterns.

Studies have indicated that the use of electronic devices such as televisions or computers that emit bright light before bedtime can adversely affect an individual’s sleep quality; with the widespread use of smartphones, this negative impact may be further exacerbated. In a questionnaire survey of 1,312 college students in Guangzhou, researchers found that students who used their phones for more than one hour before sleep and those with a higher degree of mobile phone dependence had the highest prevalence of sleep quality disorders [36]. A survey of 987 medical college students showed that the higher the degree of mobile phone dependence, the poorer the sleep quality, the shorter the sleep duration, the lower the sleep efficiency, and the more likely they were to experience sleep disorders [37]. Mobile phone dependence can significantly predict sleep [38]; mobile phone dependence directly affects sleep quality, and the greater the degree of mobile phone dependence among college students, the poorer the sleep quality [39]. Based on this, Hypothesis H3 is proposed: Mobile phone dependence will reduce sleep quality.

### The impact of mobile phone dependence on sleep duration

The Resource Allocation Theory explains how individuals balance their limited time and energy among various activities [40]. This theory is particularly applicable to exploring how individuals make choices and trade-offs between daily activities, such as physical exercise and smartphone usage. In modern society, smartphones have become an indispensable part of people’s daily lives, but excessive use, especially at night, is widely believed to have a negative impact on sleep quality. Studies have indicated that as smartphone usage time increases, individuals tend to have shorter sleep durations and later bedtimes, changes that can lead to a decline in sleep quality [41]. Mobile phone addiction has a negative effect on the sleep quality and various dimensions of college students [42]. Surveys conducted among college students show that sleep deficiency is a common problem among adolescents, and smartphone dependence is an important predictive factor for sleep insufficiency [43]. A meta-analysis of 14 studies found a significant positive correlation between adolescent smartphone dependence and sleep quality [44]. These studies emphasize the importance of a healthy lifestyle in preventing smartphone dependence and maintaining good sleep habits. Therefore, Hypothesis H4 is proposed: Mobile phone dependence has a negative impact on sleep duration. According to the Pittsburgh Sleep Quality Index (PSQI), longer sleep duration is associated with higher sleep quality; extending sleep time can improve sleep quality. Hypothesis H5 is proposed: Sleep duration can enhance sleep quality. Studies have pointed out that comprehensive intervention measures, primarily exercise, can effectively alleviate symptoms of smartphone dependence among college students and significantly improve their sleep quality [45]. With appropriate intervention measures, sleep patterns damaged by smartphone dependence can be improved [46].

**Fig. 1:**
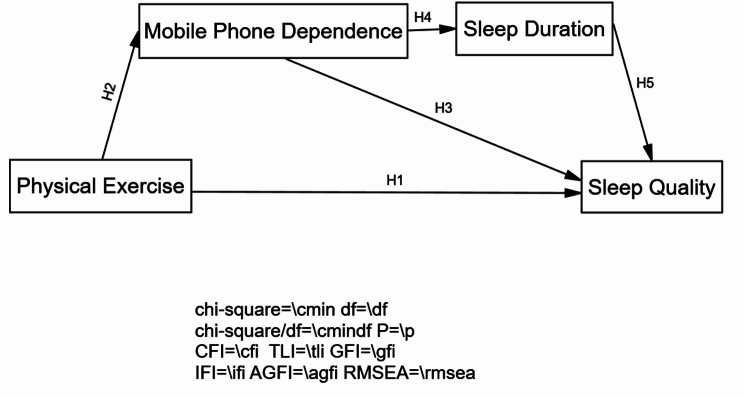
Theoretical model of mobile phone dependence and sleep duration in the relationship between physical exercise and sleep quality

In summary, physical exercise, as a positive lifestyle choice, has been proven to have potential positive effects on improving sleep quality. However, existing studies have predominantly focused on the direct relationship between physical exercise and sleep quality, with limited exploration of the underlying psychological and behavioral pathways, particularly the role of smartphone dependence. Based on this, we select four variables: physical exercise, mobile phone dependence, sleep duration, and sleep quality. By constructing a mediation effect model, we reveal the complex relationship between physical exercise and sleep quality, attempts to identify a chain mediating mechanism, provides empirical support for the design of comprehensive interventions targeting behavioral addiction and sleep physiology among college students. Based on the above hypotheses, a relationship model between physical exercise, mobile phone dependence, sleep duration, and sleep quality is constructed, as seen in Fig. 1. It is hypothesized that there is a direct effect between physical exercise and sleep quality, an indirect effect of mobile phone dependence between physical exercise and sleep quality, and a chain mediating effect of mobile phone dependence and sleep duration between physical exercise and sleep quality.

## Methods

### Data and sample

The study selected two comprehensive universities, one in the eastern part of China (Shandong Province, referred to as H) and the other in the western part (Gansu Province, referred to as G), which recruit students nationwide. The student population exhibits diversity in terms of regional and cultural backgrounds. The research subjects were limited to first and second-year university students. A cluster sampling method by class was employed, covering multiple academic fields, including Literature, History, Economics, Education, Science, Engineering, and Agriculture. Inclusion Criteria: Voluntary healthy college students over 18 years old (non-disabled) who have been continuously using smartphones in the past six months. Exclusion Criteria: Students under 18 years old and individuals unwilling to participate in the survey.

### Research tools

The assessment of physical exercise was conducted using the Physical Activity Rating Scale (PARS-3), developed by Hashimoto Kimio and revised by Liang Deqing and others from Wuhan Sports University [47]. The scale evaluates the amount of exercise from three aspects: intensity, time, and frequency of physical exercise. Exercise volume = intensity × time × frequency; the total score ranges from a minimum of 0 points to a maximum of 100 points. The Cronbach’s α coefficient for the scale in this study was 0.658. Although the Cronbach’s α coefficient was marginally below the conventional threshold of 0.70, this scale has been widely used in previous studies with Chinese populations [47].

The assessment of mobile phone dependence utilized the Mobile Phone Addiction Index (MPAI), developed by Leung and revised by Huang Hai and others [48–49]. The scale consists of 17 items and uses a Likert scoring method from 1 to 5 points. Higher scores indicate a greater degree of mobile phone dependence. The Cronbach’s *α* coefficient for the scale in this study was 0.906.

Sleep quality was assessed using the Chinese Version [50] of the Pittsburgh Sleep Quality Index (PSQI) [51]. The PSQI is commonly used to assess the sleep status of the subject over the past month, with a total score ranging from 0 to 21, and higher scores indicate poorer sleep quality. The Cronbach’s *α* coefficient for the scale in this study was 0.819.

### Measures

Online questionnaires were edited through “Survey Website” and the survey was conducted by specialized investigators. The investigators went to the research subjects’ classrooms, obtained consent from the teachers before the survey, and conducted the survey on the subjects during breaks. The investigators explained the purpose and content of the survey, used uniform guiding language, and conducted the survey anonymously. Three questionnaire links were distributed through WeChat groups, and the subjects independently completed the questionnaires online. A total of 2601 people were sent the questionnaire, and 2601 people completed it. After reviewing the quality of all the questionnaires, invalid ones (with missing items, incomplete questionnaires, and answers deviating from reality) were discarded. As a result, 1,905 valid questionnaires were obtained, with a validity rate of 73%. Among them, there were 912 students from eastern schools and 993 students from western schools, 901 males, and 1004 females.

### Statistical analysis

Data were subjected to descriptive and correlation analyses using SPSS 23.0. Structural equation modeling (SEM) was performed with AMOS 24.0 to construct chain mediation models. The assumptions of SEM were tested, including normality of variables, independence of observations, and the absence of multicollinearity. Model fit was assessed using χ²/df, CFI, GFI, AGFI, RMSEA, and SRMR. The Bootstrap Method was employed for testing the mediation effects, with 1000 resamples to calculate the 95% confidence intervals for indirect effects [52]. Statistical symbols represent the mean plus or minus the standard deviation M ± SD, and *p* < 0.05 indicates a statistically significant difference.

## Results

### Descriptive statistics and correlation analysis

The surveyed college students (*N* = 1,905) ranged in age from 18 to 21 years old, with a mean age of 19.39 years (SD = 0.86). The sample included 901 males and 1,004 females, among whom 912 students studies at H University and 993 students studies at G University. They used their mobile phones for an average of 7 h per day and spent an average of 42.22 yuan per month on mobile phone bills. There were significant correlations between physical exercise, mobile phone dependence, sleep duration, and sleep quality. The correlation coefficients are presented in Table 1. Sleep quality was inversely scored (higher PSQI scores indicate poorer quality), while sleep duration was positively scored.

**Table 1:** Descriptive statistics and correlation analysis of variables (*N* = 1905)

Variables	M ± SD	Physical Exercise	Mobile Phone Dependence	Sleep Duration	Sleep Quality
Physical Exercise	18.33 ± 19.23	1			
Mobile Phone Dependence	36.73 ± 11.95	-0.13 ***	1		
Sleep Duration	7.08 ± 0.92	0.05 *	-0.24 ***	1	
Sleep Quality	4.36 ± 2.56	-0.14 ***	0.39 ***	-0.49 ***	1

Note: * *p*<0. 05, *** *p*<0.001

### Structural equation model test

Based on the research hypotheses and the proposed conceptual model, AMOS 24.0 was utilized to establish the structural equation model to be tested. The AMOS 24.0 software employed the maximum likelihood method for parameter estimation and model fit assessment, as shown in Table 2. All major fit indices reached a good level, indicating that the overall model fit was satisfactory. The results of the hypothesis tests for all direct paths in the model are presented in Table 3, all of which were statistically significant (*p* < 0.001), suggesting that the hypotheses were reasonable. The path coefficients revealed that physical exercise negatively predicted sleep quality (higher scores indicating poorer sleep quality) and mobile phone dependence; mobile phone dependence positively predicted sleep quality and negatively predicted sleep duration.

**Table 2:** Fit indices of data and model

Fit Indices	χ^2^/df	CFI	GFI	AGFI	RMSEA	SRMR
Reference Values	≤ 3	>0.90	>0.90	>0.90	<0.08	<0.08
Test Values	0.677	1.000	1.000	0.998	<0.001	0.006

Note: χ^2^/df is the ratio of chi-square to degrees of freedom; CFI is the Comparative Fit Index; GFI is the Goodness of Fit Index; AGFI is the Adjusted Goodness of Fit Index; RMSEA is the Root Mean Square Error of Approximation; SRMR is the Standardized Root Mean Square Residual

**Table 3:** Hypothesis test results

Hypothetical Path	Standardized Path Coefficients	Non-standardized Path Coefficients	S.E.	C.*R*.	*p*	Hypothesis Results
H1:PARS3→PSQI	-0.086	-0.011	0.003	-4.500	***	Supported
H2:PARS3→MPAI	-0.130	-0.081	0.014	-5.708	***	Supported
H3:MPAI→PSQI	0.277	0.059	0.004	14.086	***	Supported
H4:MPAI→Sleep Duration	-0.243	-0.019	0.002	-10.936	***	Supported
H5:Sleep Duration→PSQI	-0.415	-1.148	0.054	-21.321	***	Supported

Note: S.E. stands for standard error; C.R. Z-value; *** *p* < 0.001, PARS3 for Physical Exercise, MPAI for Mobile Phone Dependence, PSQI for Sleep Quality

### Chain mediation effect test

**Fig. 2:**
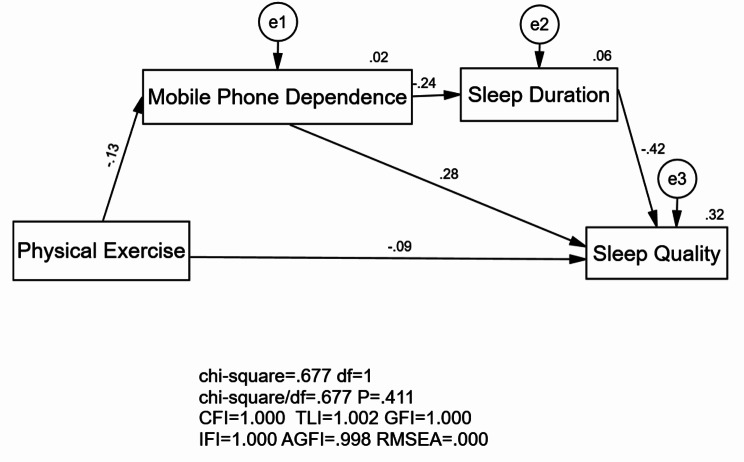
Structural equation model of mobile phone dependence and sleep duration between physical exercise and sleep quality

The Bootstrap Method was employed to test the mediating effects, with 1000 resamples used to calculate the 95% confidence intervals for the indirect effects. The total effect between physical exercise and sleep quality was − 0.135, which was composed of two pathways. The mediating effect of mobile phone dependence accounted for 27% of the total effect, and the chain mediation effect of mobile phone dependence and sleep duration accounted for 10% of the total effect. The 95% confidence intervals for these indirect effects did not include 0, indicating that both pathways were statistically significant (*p* < 0.05), suggesting that the mediating effects were significant and the pathways were established. See Table 4. Therefore, all research hypotheses were verified, and the chain mediation model is shown in Fig. 2.

**Table 4:** Mediation effect test results

Path Relationship	Standardized Effect Size	Non-standardized Effect Size	Bootstrap 1000 times 95% CI			
			Bias-Corrected		Percentile	
			Lower	Upper	Lower	Upper
Indirect Effect						
PARS3→MPAI→PSQI	-0.036	-0.005	-0.007	-0.003	-0.007	-0.003
PARS3→MPAI→Sleep Duration→PSQI	-0.013	-0.002	-0.003	-0.001	-0.003	-0.001
Direct Effect						
PARS3→PSQI	-0.086	-0.011	-0.017	-0.006	-0.017	-0.006
Total Effect						
PARS3→PSQI	-0.135	-0.018	-0.023	-0.012	-0.023	-0.012

Note: PARS3 for Physical Exercise, MPAI for Mobile Phone Dependence, PSQI for Sleep Quality

### Common method Bias test

To ensure the reliability of the research findings, a common method bias test was conducted. Harman’s single-factor test method was utilized to perform an exploratory factor analysis on all scale items. During the analysis, it was found that there were 9 factors with eigenvalues greater than 1. The factor with the highest explanatory power accounted for 21.89% of the total variance, a proportion that is below the critical threshold of 40% [53]. This indicates that the issue of common method bias is not severe.

## Discussion

### The impact of physical exercise on sleep quality

The results suggest that physical exercise can directly and indirectly predict sleep quality, and as an effective health intervention, physical exercise can improve sleep quality. This finding is consistent with existing literature, with studies suggesting that college students with regular exercise habits have better sleep [54]. Adolescents who frequently engage in physical activities show better sleep quality compared to their sedentary counterparts [55]; comprehensive exercise can improve the subjective and objective sleep quality of college students with sleep disorders and reduce the incidence of sleep disorders [56]. A meta-analysis of exercise interventions on the sleep of 670 adult subjects suggests that exercise interventions tend to improve sleep efficiency [57]. The observed effects may operate through neurobiological mechanisms-exercise-induced enhancement of cerebral blood flow and BDNF expression potentially counteracts smartphone-related cognitive hyperarousal, facilitating sleep initiation. College students often face academic and life pressures, and physical activity can help them release stress and improve sleep quality [58]. Schools should encourage and design more accessible physical exercise programs to promote a healthy lifestyle among students; this can be achieved by increasing the diversity of physical education courses, providing more sports facilities, and hosting campus sports events to spark students’ interest in exercise. The impact of physical exercise on sleep quality is a multidimensional research field involving physiological, psychological, and social aspects. However, the timing, intensity, and type of exercise also affect its impact on sleep, which requires further evidence.

### The sole mediating effect of mobile phone dependence

The study further reveals through mediation analysis that physical exercise may indirectly improve sleep quality by reducing mobile phone dependence. It has been observed in various studies that adolescents are more susceptible to mobile phone addiction [59]. With the proliferation of smartphones, their multifunctionality and convenience have led to a high level of dependence among college students. This dependence not only affects their daily lives but may also have a negative impact on their sleep quality. Excessive use of smartphones and other electronic devices before bedtime can over-stimulate our brains, which is particularly common among many college students. This sustained state of brain activity is not conducive to entering the necessary restful state, potentially affecting the quality and depth of sleep [39]. Further research has indicated that improper mobile phone usage habits and excessive reliance on phones have a negative impact on the sleep health of college students [60]. The environmental factor of mobile phone use as it affects sleep is gaining increasing attention from scholars. Therefore, by increasing physical exercise among college students, it can effectively reduce mobile phone usage time, help individuals establish healthier living habits, and thereby improve sleep quality. Academic and recreational smartphone uses may overlap, complicating dependence interpretations. Future research should distinguish usage patterns to clarify their impacts.

### The chain mediating effect of mobile phone dependence and sleep duration

The study explored the chain mediating role of mobile phone dependence and sleep duration between This study advances the literature by demonstrating a novel chain mediation pathway. Unlike prior studies focusing on single mediators, our model integrates behavioral (mobile phone use) and physiological (sleep duration) mechanisms, offering a holistic framework for interventions. Surveys have shown that stress perception and mobile phone addiction play a chain mediating role between physical exercise and sleep quality [61]. According to other research findings, when college students shift from no physical exercise to moderate-intensity exercise, their tendency towards mobile phone dependence significantly decreases. This indicates that engaging in physical activities can effectively reduce the dependency of college students on mobile phones. In particular, moderate-intensity physical activities have a significant positive effect on improving the phenomenon of mobile phone dependence among college students [62]. Smartphones are not only a means of communication but also an indispensable part of college students’ personal social and work lives. Apart from necessary information acquisition, mobile phone chats and entertainment occupy a lot of time, and playing with mobile phones is almost a lifestyle as long as they are not sleeping. This may lead to reduced sleep duration and lower sleep quality. Partial mediation suggests physical exercise improves sleep quality through direct (e.g., physiological regulation) and indirect pathways (e.g., reducing dependence). This highlights the need for multifaceted interventions targeting multiple mechanisms. These findings provide a new perspective on how physical exercise promotes sleep quality and are of significant importance for designing targeted intervention measures. In the college student population, increasing physical exercise can improve sleep quality.

While the study provides valuable insights, several limitations should be acknowledged. The research relied on self-reported questionnaires administered to college students, a methodology vulnerable to reporting bias. Specifically, sleep duration and quality assessments using the Pittsburgh Sleep Quality Index (PSQI) depend on participants’ subjective recall, which may introduce measurement inaccuracies due to recall bias [63]. Although the PSQI remains a practical tool, future investigations would benefit from integrating objective measures (e.g., actigraphy) with self-report instruments to improve the validity of sleep quality evaluations. A limitation of this study is the relatively low reliability of the PARS-3 scale (α = 0.658), which may reduce the precision of physical exercise measurement. Future studies could consider using more validated instruments. Additionally, the cross-sectional design of the study limits the in-depth analysis of causal relationships. To more accurately reveal the long-term effects between variables, future studies could employ longitudinal tracking design methods.

## Conclusion

By analyzing the survey data from 1,905 college students, the study explored the relationship between physical exercise, mobile phone dependence, sleep duration, and sleep quality. A significant positive relationship was found between physical exercise and sleep quality, which aligns with existing literature that emphasizes the potential value of physical exercise in promoting healthy sleep. Mobile phone dependence acted as a mediator between physical exercise and sleep quality, providing a new perspective on how physical exercise may indirectly improve sleep quality by reducing mobile phone dependence. The mediation effect was partial, with both direct and indirect paths contributing to the relationship between physical exercise and sleep quality. Based on these findings, it is recommended to promote more physical activities among college students to reduce mobile phone dependence and improve sleep quality. This approach not only directly enhances sleep quality but also indirectly improves it by reducing mobile phone dependence and increasing sleep duration.

## Data Availability

The original contributions presented in the study are included in the article material, further inquiries can be directed to the corresponding author.
